# Supplementary material for: Estimating the global demand curve for a leishmaniasis vaccine: A generalisable approach based on global burden of disease estimates
Source: PLoS Negl Trop Dis. 2022 Jun 13;16(6):e0010471. doi: 10.1371/journal.pntd.0010471 (PMC9232160; doi:10.1371/journal.pntd.0010471)
Supplement: S1 Table — (DOCX) [file pntd.0010471.s001.docx]

S1 Table: Vaccine rollout projection (2030-2040)

|  | **Number of people vaccinated between 2030 and 2040 (for CL prevention)** | | | **Number of people vaccinated between 2030 and 2040 (for VL prevention)** | | |
| --- | --- | --- | --- | --- | --- | --- |
| **Country** | **0-4 years** | **5-14 years** | **15-29 years** | **0-4 years** | **5-14 years** | **15-29 years** |
| Afghanistan | 2,749,122 | 5,885,515 | 4,328,652 | - | - | - |
| Algeria | 1,957,406 | 5,163,208 | 2,047,082 | - | - | - |
| Bangladesh | - | - | - | 3,258,918 | 8,191,346 | - |
| Brazil | - | - | - | 10,502,595 | 23,450,987 | - |
| China | - | - | - | 23,028,828 | 53,370,973 | - |
| Ethiopia | 1,812,940 | 2,876,160 | 1,900,381 | 1,138,038 | 1,805,454 | - |
| Georgia | - | - | - | 309,726 | 626,678 | - |
| India | 20,186,202 | 40,113,892 | 27,707,496 | 14,573,985 | 32,893,737 | - |
| Israel | 2,173,296 | 4,419,907 | 2,299,880 | - | - | - |
| Kenya | - | - | - | 1,182,557 | 2,186,535 | - |
| Morocco | 1,267,528 | 2,450,203 | 1,160,361 | - | - | - |
| Nepal | - | - | - | 3,293,786 | 8,223,698 | - |
| Nigeria | 1,305,681 | 2,560,543 | 1,149,318 | - | - | - |
| Pakistan | 22,071,512 | 49,465,950 | 29,566,359 | - | - | - |
| Paraguay | - | - | - | 642,108 | 1,436,384 | - |
| Saudi Arabia | 643,958 | 1,558,356 | 922,762 | - | - | - |
| Somalia | - | - | - | 513,476 | 1,071,733 | - |
| South Sudan | - | - | - | 305,306 | 743,962 | - |
| Spain | - | - | - | 3,059,899 | 7,111,607 | - |
| Sudan | 15,699,043 | 20,950,603 | 13,581,912 | 2,028,979 | 3,452,805 | - |
| Syria | 4,732,334 | 11,634,572 | 5,266,336 | - | - | - |
| Tunisia | 981,419 | 2,287,679 | 1,178,702 | - | - | - |
| Turkey | 7,182,351 | 11,545,177 | 5,136,730 | - | - | - |
| Uzbekistan | 3,378,644 | 8,001,227 | 4,350,362 | - | - | - |
| Total | **564,054,859** | | | | | |

Source: Malvolti S, Malhame M, Mantel C, Rutte EA Le, Kaye PM. Human leishmaniasis vaccines: use cases, target population and potential global demand. PLoS Negl Trop Dis. 2021;15(9):e0009742. Available from: https://doi.org/10.1371/journal.pntd.0009742
