# Supplementary material for: Estimating the global demand curve for a leishmaniasis vaccine: A generalisable approach based on global burden of disease estimates
Source: PLoS Negl Trop Dis. 2022 Jun 13;16(6):e0010471. doi: 10.1371/journal.pntd.0010471 (PMC9232160; doi:10.1371/journal.pntd.0010471)
Supplement: S3 Table — (DOCX) [file pntd.0010471.s003.docx]

S3 Table: Projected GAVI support status in 2030

| **Country** | **Projected country support status in 2030** |
| --- | --- |
| Afghanistan | Initial self-financing |
| Algeria | Not eligible |
| Bangladesh | Preparatory transition |
| Brazil | Not eligible |
| China | Not eligible |
| Ethiopia | Preparatory transition |
| Georgia | Not eligible |
| India | Accelerated transition |
| Israel | Not eligible |
| Kenya | Accelerated transition |
| Morocco | Not eligible |
| Nepal | Preparatory transition |
| Nigeria | Accelerated transition |
| Pakistan | Accelerated transition |
| Paraguay | Not eligible |
| Saudi Arabia | Not eligible |
| Somalia | Initial self-financing |
| South Sudan | Initial self-financing |
| Spain | Not eligible |
| Sudan | Not eligible |
| Syria | Preparatory transition |
| Tunisia | Not eligible |
| Turkey | Not eligible |
| Uzbekistan | Not eligible |
