# Supplementary material for: Estimating the global demand curve for a leishmaniasis vaccine: A generalisable approach based on global burden of disease estimates
Source: PLoS Negl Trop Dis. 2022 Jun 13;16(6):e0010471. doi: 10.1371/journal.pntd.0010471 (PMC9232160; doi:10.1371/journal.pntd.0010471)
Supplement: Attachment — Submitted filename: Revision_responses_FINAL.docx [file pntd.0010471.s004.docx]

**Subject:** Submission of responses to Reviewer comments on submitted manuscript

**Title:** Estimating the global demand curve for a leishmaniasis vaccines: a generalisable approach based upon global burden of disease estimates.

Dear Editor,

We would like to submit our revision of the paper entitled “**Estimating the global demand curve for a leishmaniasis vaccines: a generalisable approach based upon global burden of disease estimates**”. We are grateful to the reviewers who have offered valuable insights and suggestions. We provide our responses to the reviewer report, below.

Yours truly (On behalf of the authors),

Sakshi Mohan,

Centre for Health Economics, University of York.

## **Methods**

-Are the objectives of the study clearly articulated with a clear testable hypothesis stated?
-Is the study design appropriate to address the stated objectives?
-Is the population clearly described and appropriate for the hypothesis being tested?
-Is the sample size sufficient to ensure adequate power to address the hypothesis being tested?
-Were correct statistical analysis used to support conclusions?
-Are there concerns about ethical or regulatory requirements being met?

Reviewer #1:

**1.1** Methods and underlying assumptions and sensitivity analyses are well described. This is a generalised approach that recognises data limitations but, as such, does provide a foundational piece of work that can be adapted for diseases other than VL/CL.  The sample size of selected countries based on overall VL/CL burden seems appropriate. Overall developmental need for the analysis is well described and the work seeks top bridge information gaps between demand, supply and possible value price points for new products (in this case a vaccine for VL/CL).

Thank you for the positive feedback.

Reviewer #2:

**2.1** As for the model itself would encourage the authors to adopt a **probabilistic approach** in which all 14 input factors are characterized by their probability distribution function (pdfs) and the model is run in a Monte Carlo approach (see Saltelli A (2008)). Yet, model output can be characterized probabilistically by accounting for the full variability and uncertainty of values. If averages are provided I would feel much more comfortable
In any event, overall it would be nice to see some pdfs of epitomic countries and maps of countries color-coded based on incidence under different scenarios. I believe tables are hard to read and communicate little. They can be placed as SI.

Whilst we recognize the value of models being probabilistic to support decisions, and indeed this is the approach we would take in much of our work, this has been challenging to do for this analysis. We list the data inputs used in the table below and details on why probabilistic analysis has not been possible in our study. However, we have sought to better reflect the extent of uncertainty through providing lower- and upper-bound estimates of prices, shown in Figures 1-3 and Tables 3-4, as described below.

| **Input** | **Whether uncertainty captured** | **Why uncertainty could not be captured/ How uncertainty was captured** |
| --- | --- | --- |
| 1. Cost-effectiveness Threshold (CET) | No | These estimates were taken from Lomas et al. (2021) (Lomas et al., 2021). The authors only provide point estimates for CET projections, stating that further research is required to capture parameter uncertainty for future values.  For 7 countries (Afghanistan, Israel, Saudi Arabia, Somalia, South Sudan, Spain, Syria), CET estimates were based on Ochalek et al. (2020) which expresses CET as a generic percentage of GDP per capita for each of the four World Bank Income levels. While it would be possible to apply the range of GDP per capita projections for these estimates, we did not think it would be consistent to apply uncertainty to only these 7 countries. |
| 2. CL/VL Incidence $(I)$ | Yes | These estimates were taken from the Global Burden of Disease (GBD) study, 2019 (Institute for Health Metrics and Evaluation, 2020). The GBD study provides lower and upper bound estimates (i.e. a range) for incidence and burden of disease. Since probability distribution functions (pdfs) are not provided, we were unable to capture the uncertainty around these inputs probabilistically but rather incorporated the range of values in our analysis by showing the upper and lower bounds of value-based maximum price payable by countries under the two extreme scenarios as well as the point estimates provided by GBD. (see Table 3-4, Figure 1-3) |
| 3. CL/VL DALY burden $(DALY)$ | Yes |  |
| 4. Treatment cost (${T\_pp}_{\alpha})$ | No | The studies from which CL and VL treatment costs were extracted did not provide uncertainty estimates (de Carvalho et al., 2017; Eid Rodríguez et al., 2019). These were chosen because they were some of the few studies estimating comprehensive direct costs including the cost of staff time, ambulatory care, hospitalisation, etc. Whereas we could have supposed probability distributions over these costs, we felt this would have been too speculative. |
| 5. Treatment coverage $(\theta$) | Yes | In the absence of data on leishmaniasis treatment coverage, we project the value-based maximum price under the assumptions of both 0% and 100% treatment coverage to represent its upper and lower bounds.  Two separate demand curves are provided, one assuming no treatment coverage and another 100% treatment coverage. We chose not to integrate these two within the same figure so that the effect of difference in treatment coverage would not be masked by the variation in value-based maximum price due to changes to vaccine efficacy and uncertainty around disease incidence and burden, which account for a majority of the variation in price. |
| 6. Discount rate $(r)$ | No | The standard rate of 3% was applied. Discount rate represents time preference rather than something which there is sampling uncertainty, so is not typically included probabilistically. |
| 7. Quantity Demanded $(q)$ | No | These projected estimates of the number of vaccines which can be rolled out each year were obtained from Malvolti et al. (Malvolti et al., 2021), which only provides point estimates on vaccine demand. |
| 8. Number of years of efficacy $(N)$ | No | Rather than making the results harder to interpret by allowing for variation in the duration of efficacy, we chose to update our initial analysis that assumed 10 years of efficacy to a more conservative assumption of 5 years of efficacy, based on the following conservative assumption from Malvolti et al. (2021):  “A minimum of five years duration of protection was assumed based on the consideration that a shorter duration would likely render the vaccine too cumbersome for implementation and too expensive for use in view of the number of doses required to maintain protection for the first 15 years of life (e.g., the age groups with the highest disease burden).” (Malvolti et al., 2021)  This assumption corresponds to the assumption made in one of two papers which look at the cost-effectiveness of a leishmaniasis vaccine for humans (Bacon et al., 2013). The second study (Lee et al., 2012) does not state the assumption on duration of efficacy. Whereas this could have been included probabilistically, to run the analysis for this input alone we felt would have been of limited value. We believe this assumption to be particularly conservative, but it aligns with our central aim to assess and demonstrate the commercial viability and public health value of a vaccine if found to be effective in Phase III trials. |
| 9. Vaccine efficacy | Yes | We considered a range of efficacy profiles (50-95%) with 75% efficacy representing the assumed efficacy for the point estimates.  This assumption corresponds to the efficacy profile assumed in one of two papers which look at the cost- effectiveness of a leishmaniasis vaccine for humans (Bacon et al., 2013). The second study (Lee et al., 2012) considers efficacy as low as 25% but such a low efficacy was not considered in our study since the minimum efficacy considered by Malvolti et al. (2021) in generating their demand estimates was 50% (Malvolti et al., 2021). Given these are purely assumptions, rather than subject to sampling uncertainty as such at this point, we believe the interpretation of results is clearer by including these deterministically. |

Please note that the above inputs represent all of the variable inputs in the calculation of the value-based maximum price of the vaccine, since some of the 14 items listed under box 1 are simply identifiers – i = country, β = age group, n = year, t = year of vaccination, $\alpha=VL/CL.$

**2.2** If possible I believe it would better to present results in a portfolio approach, that is **total incidence reduction vs. resources needed or value-based maximum price, rather than price as a function of demand**. I believe a population outcome should be the main evaluative outcome and not the value-based maximum price. Efficiency frontiers can be determined and displayed as a set of points and each point has different efficiency. it is **hard to imagine that efficiency and treatment coverage is constant** for so many solutions determined by demand and price (although theoretically possible).

Thank you for this suggestion, which we believe would be useful for some purposes – for instance, to guide international commitments to a new or existing global fund to support leishmaniasis vaccine purchase. However, our intention in this paper has been to demonstrate the commercial viability and maximum ability to pay of countries in committing their own health care resources, recognizing there are other things their health care budgets could be used for. This has required estimates of the marginal productivity of their health systems, i.e., the cost needed to be incurred by the health system to avert a single DALY, which is a measure of the health opportunity costs associated with committing national resources to leishmaniasis vaccine purchase. Of course, this means ability to pay differs widely across countries, but is reflective of the choices they face and the estimated demand curves reflect this.

On the demand side, whilst assuming linearity in efficiency across different levels of treatment coverage is a highly simplified case, we thought that it was beyond the scope of our paper to account for (dis)economies of scale/scope in vaccine rollout which could change the maximum ability to pay for vaccine courses by quantity demanded within each country’s total demand. This is also challenging because efficiency would depend on the vaccine rollout strategy adopted by each country, for example whether VL/CL immunisation is combined with a parallel immunization effort, alternative strategies available including treatment and vector management. Our paper simply provides the average value-based maximum price payable by a country per person at risk vaccinated (including delivery costs).

On the cost side, the notion of an average unit production cost is a reasonable one we believe for production planning. The marginal unit cost is likely to be subject to economies (or perhaps diseconomies) of scale, but our prices provide the envelope within which actual production costs can be determined.

**2.3** The probabilistic approach is also very useful for a **non-linear sensitivity analysis that can be carried out even by using a variance-based approach but considering all input factors as changing together (see Pianosi et al (2016)).** At the moment the authors used only a simple linear sensitivity approach on two parameters to alter vs. 14 input factors, as well as the neglected non-linear interactions to define which factor is truly altering model outputs. Of course later on you can analyze model output dependent on efficiency and treatment coverage but a-priori you cannot know whether these factors are the most important one (additionally these factors are non-linearly dependent with each other).

Please see the responses to 2.1 and 2.2. In the revised manuscript we have tried to show the extent of uncertainty in epidemiological factors (disease incidence and burden) and vaccine efficacy more clearly, treating the two as independent inputs in the determination of value-based maximum price payable. While there could be some dependency between these two inputs, it is unclear at this stage what this dependency could be and so we felt this to be beyond the scope of the current study.

**2.4** Lastly, I am not sure why the **axes of the graph is cut** ...

The y-axis of the graphs was broken in order to visualise the demand curve more closely for a majority of the countries for which the value-based maximum price was under $10. For example, Figure 1.i would look as follows without creating a discontinuous y-axis; a majority of the countries’ value-based maximum price payable, including the median value-based maximum price, lies within the part of the curve which appears to be flat. We therefore cut the y-axis to better convey the information contained.

Reviewer #3:

The objective of providing a simple framework for estimating vaccine affordability and testing it in a range of countries for Leishmaniasis is clearly stated.

**3.1** There is no test of the validity of the estimates which is especially important as the authors suggest using the methodology as a general framework. The authors state that more complex models exist for some countries for their use-case but there is no comparison done of the estimates from the simplified framework to more complex ones making an assessment of the quality and validity difficult.

Other studies (Bacon et al., 2013; Lee et al., 2012) have tried to assess the cost-effectiveness of a potential leishmaniasis vaccine under variable efficacy profiles and price points. Lee et al. (2012) use a Markov decision analytic computer simulation model to “(evaluate) the economic value of vaccination from a societal perspective and compared the cost-effectiveness of vaccination in Bihar, India of two different types of available treatments for VL: (1) standard amphotericin B or (2) liposomal amphotericin drug treatments.” Bacon et al. (2013) use a similar method to “evaluate the potential economic value of a preventative CL vaccine in seven countries in Latin America: Bolivia, Brazil, Colombia, Ecuador, Mexico, Peru, and Venezuela”.

These studies assess the cost-effectiveness of the vaccine at a given price and account for alternative strategies (such as treatment). The results of these studies are as follows:

- “Cost savings resulted when vaccination cost was ≤$5 regardless of vaccine efficacy; vaccination continued to avert costs when vaccination was ≤$30 and at least 50% efficacious” (Lee et al., 2010)**.** However, cost-effectiveness is assessed assuming a cost-effectiveness threshold of 1XGDP per capita rather than the marginal productivity of the health system (Lomas et al., 2022) that we use, so comparability is limited.
- A vaccine with the baseline vaccine profile (2-dose, $0.5/dose, 70% vaccine efficacy, 5 year protection duration) required 0.1% infection risk to cost less to avert a case than the cost of treatment with pentavalent antimonials ($180). Use of a $5/dose vaccine does not save costs unless CL risk resembled an epidemic (i.e., 5%). This is a cost-benefit analysis comparing vaccination costs to treatment costs (Bacon et al., 2013), so again is an alternative methodological approach.

Due to the differences in methodology and intent of the papers, we did not comment extensively in the manuscript. However, while are results are not directly comparable to either of these studies because of the difference in approach, our result that India would be able to pay between $3.5-3.8 is comparable to the result from Lee et al. (2010). The slightly more optimistic result in Lee et al. (2010) can be attributed to the higher incidence assumed – 0.5% - 3.4% depending on the age group. In our study, we assume a VL incidence of 0.4-1.7% in India. Despite the challenges in comparability, we therefore believe our results are at least not inconsistent with these studies.

**3.2** The authors do not include uncertainty in the used parameters but provide point estimates without CIs. As there is substantial uncertainty in a lot of the used data especially from GBD it is absolutely necessary to include those. Point estimates alone are uninformative.

Please see response to 2.1. Unfortunately, probability distributions in the GBD estimates are not available, but we now present our results with wide uncertainty bounds.

**3.3** The results are strongly dependent on the chosen efficacy for the vaccine which is clear from both the formulas and the results. As such I don't consider varying the efficacy a sensitivity analysis as we know it's very sensitive to it but rather complete results should be shown for a range of values.

Please see response to 2.1. We agree with the Reviewer on this point. Figures 1-3 and Tables 3-4 have been updated so that a range of efficacy assumptions is applied to capture uncertainty around our estimates under the base scenario as well as sensitivity analyses.

Reviewer #4: Yes to all of the above; no concerns

**Results**
-Does the analysis presented match the analysis plan?
-Are the results clearly and completely presented?
-Are the figures (Tables, Images) of sufficient quality for clarity?

Reviewer #1: Yes

Reviewer #2:

**2.5** Results should also consider non-linearity in model factors (for sensitivity analysis)

Please see response to 2.2 on why non-linearity in costs/efficiency was not incorporated.

Reviewer #3:

**3.4** The result section is very short and numbers are given with too many digits in the table which gives an impression of certainty that is not warranted. All results need CIs as mentioned in previous comments.

Please see updates to tables 3 and 4. We rounded the prices to 2 decimal points because the value-based maximum price for some countries was lower than $0.1.

Reviewer #4:

**4.1** Yes to the first two items. The figures I received were a bit low-resolution and showed artefacts (horizonal smears) at the breakpoints; this has been noted in the request for minor revisions.

Thank you for pointing this out - the figures have now been updated.

**Conclusions**
-Are the conclusions supported by the data presented?
-Are the limitations of analysis clearly described?
-Do the authors discuss how these data can be helpful to advance our understanding of the topic under study?
-Is public health relevance addressed?

Reviewer #1

Yes. Limitations in terms of data are described. Underlying assumptions are described with underpinning rationale.

Thank you for the positive feedback.

Reviewer #2:
**2.6** The manuscript ''Estimating the global demand curve for a leishmaniasis vaccine: a generalisable approach based on global burden of disease estimates'' is interesting and fit the journal. While I do not have major concerns about the topic or the results (if I just focus on the correctness of what has been done) I question some conceptual arguments and the sensitivity analysis of the model. I believe theoretically the paper has its own validity but not rather practically since it neglects many factors such as **multiple interventions, countries and diseases**, whose **synergies** with leishmaniasis is fundamental for assessing the demand and effectiveness curve for a leishmaniasis vaccine.

We appreciate the Reviewer’s recognition of the value of the approach we put forward. The task we set ourselves was to be informative even with very limited data availability and analytical capacity (i.e., in the absence of context-specific leishmaniasis disease models, which could capture interactions between **multiple interventions,** since the product development investment decisions still need to be made in the absence of these. Our intention has therefore been to provide preliminary and directional analyses to guide further debate and deliberation on investment decisions.

By using estimates of the marginal productivity of the health system to define cost-effectiveness thresholds to determine country-level ability to pay, we believe that we have accounted for other competing **disease** priorities which might be faced by countries, given current information (please see response to 2.7). Finally, whereas interactions across **countries** are possible, and are highly important for many neglected tropical diseases, we think that country interdependencies relevant to leishmaniasis are likely to be less given that populations at risk are usually highly localised. Nevertheless, we would anticipate that our analyses support global availability of leishmaniasis vaccine, so we don’t feel we have excluded significant externalities across borders – but if they were to exist, if anything, they would increase the maximum price in any one country (if there were concern for such externalities).

I do like the incidence-economic evaluation approach however, I think the authors should discuss the limitations of their study that as it is a modeling exercise only without the consideration of other factors to make it applicable. 
Whenever an action is evaluated, there should be the recognition of other potential interventions that aim to decrease disease incidence (see Chan LYH et al (2021) in a portfolio approach; the case study was done for COVID and NPI but it can be applied to any disease and intervention). For leishmaniasis other factors are possible such as ecosystem management, targeting either habitats (ecohydrological controls) and specific species as animal hosts (Servadio J et al., (2020)), leaving aside education campaigns.

There is only one vaccine contender currently and our assumptions of 0% and 100% treatment availability reflect the extremes of treatment access. Whereas interactions with ecological interventions and other (non-leishmaniasis) interventions are possible, we believe it would be highly speculative to include anything other than the current burden of disease from leishmaniasis (which, it should be noted, is the current burden even with the presence and use of other existing interventions). We do agree with the point, however, that investment decisions should be guided by consideration of ‘all relevant comparators’, including those ecosystem management and educational campaigns described, so have highlighted this in the Discussion. Thank you for the suggestion.

**2.7** Secondly, at the world and country scale, other diseases should be considered when prioritizing a vaccine. This is the third element that is neglected and then should be discussed.

The marginal productivity estimates that we use to determine the maximum prices reflect the opportunity costs that health systems would incur in committing budgets to provision of leishmaniasis vaccine, rather than to other alternative forms of health care they could otherwise fund (including other vaccines and therapeutics). As such, consideration of the funding claims represented by other interventions is already integral to the approach we have set out.

**2.8** Lastly, country interdependencies is extremely important when evaluating the effectiveness of a vaccine because boundaries are less and less important in disease transmission but also in terms of vaccine development decision (at the world scale). 
Certainly the consideration of all these factors is hard but doable. It is ok not to include these features in this paper BUT I believe these three elements must be discussed because a vaccine cannot just be evaluated by considering leishmaniasis by itself country by country.

Thank you for these suggestions. We have added to the Discussion (lines 354-361) to reflect these points.

Reviewer #3:

**3.5** The authors state that their framework overcomes the challenges of a lack of detailed data for a full epidemiological model by making **simplifying assumptions**. There is no clear discussion of what those assumptions are and how they might affect the estimates.

We have added to the Discussion to outline these assumptions more clearly (lines 354-361). In essence, these assumptions relate to the absence of context-specific infectious disease models available for all the countries which are likely to represent the biggest markets for a leishmaniasis vaccine, if it were to become available. This limitation is not likely to be overcome anytime soon, for leishmaniasis or many other diseases, but development decisions still need to be made. This was the main motivation to develop and apply a simpler, admittedly limited but generalizable approach. We have now outlined this motivation more fully in the Discussion.

**3.6** Also a discussion of how uncertainty in the data that is used even for the simplified framework might affect the usefulness of the estimates is needed. Currently this topic seems to have been avoided in the manuscript.

We have made efforts to further reflect the key uncertainties, although this remains somewhat limited for the reasons outlined in response to comments 2.1.

Reviewer #4: Yes to all, no concerns.

Thank you.

Editorial and Data Presentation Modifications?
Use this section for editorial suggestions as well as relatively minor modifications of existing data that would enhance clarity. If the only modifications needed are minor and/or editorial, you may wish to recommend “Minor Revision” or “Accept”.

Reviewer #2:

**2.9** Data should be better presented as highlighted in my review of results

Thank you – we have made this change.

Reviewer #4:

**4.2** General: You do not state the software in which this analysis was conducted, nor do you describe the public availability of code that would make this analysis truly replicable. Please list software names and versions, and either link to a public code repository, include your code in the supplementary materials, or explain why you are not able to share your code.

We have now added this to the manuscript text: All the analyses were performed on excel and figures were produced on Python. The workbook and code are publicly available on [GitHub](https://github.com/sakshimohan/leish-vaccine). (Please see lines 273-274).

## **Further editorial changes:**

**Text:**
1. grammatical note: please leave a space between words and citations.
2. line 111: "is" endemic, not "in" endemic

3. use either "Gavi" or "GAVI" throughout the document.

Thank you for these editorial recommendations. We have incorporated these suggestions.

4. a brief description of what sort of disease leishmaniasis is (what are the vectors, etc) would strengthen the introduction's discussion of different control options.
Some further detail on leishmaniasis has been provided in the Introduction (lines 66-70). There is also information on the disease in lines 80-91.

5. lines 88-94: can you cite literature to support the importance of these two stakeholder groups in vaccine development?

Reference added in line 95

6. lines 129-131: state why the countries representing the other 20% of burden were excluded.

The unavailability of data on the population at risk and vaccine coverage projections from Malvolti et al (2021) was the reason for exclusion of the other countries with the remaining 20% burden. (Please see lines 135-137)

7. In the discussion, please also note the limitation that GBD estimates themselves are modeled and highly uncertain, especially in countries with little or no data.

Limitation added in lines 361-365.

8. If the COVID-19 pandemic has any potential impact on the results or implications of this analysis, please state them.

Although there is some evidence that COVID-19 has disrupted prevention and treatment for a wide range of diseases, it is not known what the implications have been for leishmaniasis, but the magnitude is likely to be relatively modest and it is unclear how long the effects of disruptions will be for leishmaniasis or other diseases, so we have not made any statements in the manuscript on this.

**Figures and tables, etc:**
1. Box 1: please include units for all definitions.

Units have now been included in Box 1 (Please see line 249).

2. Figures: In the versions of the images I received, there are strange artefacts (smeared horizontal lines) at the breakpoints of the plots. Please ensure these are removed prior to resubmission.

Figures verified with PACE.

3. Figures 2-4 would be more effective if you also included the comparison curve from Figure 1 on the same plot-- it is difficult to compare otherwise.

We agree with the reviewers. This change has been made to figures 2-3.

**Further changes**

1. Instead of emphasising the median value-based maximum price in the results, we now focus on the average value-based maximum price, weighted by quantity demanded, because the former skewed the result towards countries representing a smaller proportion of the market (lines 36-37, 60-61, 284-286, 302-303, 308-312, 335-336).

## **References**

Bacon, K. M., Hotez, P. J., Kruchten, S. D., Kamhawi, S., Bottazzi, M. E., Valenzuela, J. G., & Lee, B. Y. (2013). The potential economic value of a cutaneous leishmaniasis vaccine in seven endemic countries in the Americas. *Vaccine*, *31*(3), 480–486. https://doi.org/10.1016/J.VACCINE.2012.11.032

de Carvalho, I. P. S. F., Peixoto, H. M., Romero, G. A. S., & de Oliveira, M. R. F. (2017). Cost of visceral leishmaniasis care in Brazil. *Tropical Medicine & International Health*, *22*(12), 1579–1589. https://doi.org/10.1111/tmi.12994

Eid Rodríguez, D., San Sebastian, M., & Pulkki-Brännström, A.-M. (2019). “Cheaper and better”: Societal cost savings and budget impact of changing from systemic to intralesional pentavalent antimonials as the first-line treatment for cutaneous leishmaniasis in Bolivia. *PLOS Neglected Tropical Diseases*, *13*(11), e0007788. https://doi.org/10.1371/journal.pntd.0007788

Institute for Health Metrics and Evaluation. (2020). *Global Burden of Disease Study 2019 (GBD 2019) Data Resources*. IHME. http://ghdx.healthdata.org/gbd-2019

Lee, B. Y., Bacon, K. M., Connor, D. L., Willig, A. M., & Bailey, R. R. (2010). The Potential Economic Value of a Trypanosoma cruzi (Chagas Disease) Vaccine in Latin America. *PLoS Neglected Tropical Diseases*, *4*(12), e916. https://doi.org/10.1371/journal.pntd.0000916

Lee, B. Y., Bacon, K. M., Shah, M., Kitchen, S. B., Connor, D. L., & Slayton, R. B. (2012). The economic value of a visceral leishmaniasis vaccine in Bihar State, India. *American Journal of Tropical Medicine and Hygiene*, *86*(3), 417–425. https://doi.org/10.4269/ajtmh.2012.10-0415

Lomas, J., Claxton, K., & Ochalek, J. (2021). Accounting for country- and time-specific values in the economic evaluation of health-related projects relevant to low- and middle-income countries. *Health Policy and Planning*, *00*, 1–10. https://doi.org/10.1093/HEAPOL/CZAB104

Lomas, J., Claxton, K., & Ochalek, J. (2022). Accounting for country- and time-specific values in the economic evaluation of health-related projects relevant to low- and middle-income countries. *Health Policy and Planning*, *37*(1), 45–54. https://doi.org/10.1093/HEAPOL/CZAB104

Malvolti, S., Malhame, M., Mantel, C., Rutte, E. A. Le, & Kaye, P. M. (2021). Human leishmaniasis vaccines: use cases, target population and potential global demand. *PLoS Neglected Tropical Diseases*, Forthcoming. https://doi.org/forthcoming

Ochalek, J., Claxton, K., Lomas, J., & Thompson, K. M. (2020). Valuing health outcomes: developing better defaults based on health opportunity costs. *Https://Doi.Org/10.1080/14737167.2020.1812387*. https://doi.org/10.1080/14737167.2020.1812387
